# Synthesis of Nanocomposites and Catalysis Applications II

**DOI:** 10.3390/nano13233054

**Published:** 2023-11-30

**Authors:** Evgeny Gerasimov

**Affiliations:** Boreskov Institute of Catalysis SB RAS, 630090 Novosibirsk, Russia; gerasimov@catalysis.ru

Nanocomposites, which refer to materials composed of nanoparticles dispersed in a matrix, have gained significant attention in various fields due to their unique properties and potential applications. One important area where nanocomposites have found extensive use is in catalysis. In this regard, nanocomposites can be tailored to enhance catalytic activity, selectivity, and stability, making them promising candidates for numerous catalytic applications. This is especially noticeable in various reactions of hydrocarbon transformations [1,2].

Once the nanocomposite is fabricated, it can be used for various catalytic processes. For example, in heterogeneous catalysis, the nanocomposite can act as a catalyst for multiple reactions, including oxidation, reduction, hydrogenation, and dehydrogenation. The presence of nanoparticles in the nanocomposite increases the surface area, leading to improved catalytic activity. Moreover, the composition and properties of the nanoparticles can be precisely controlled, allowing for the design of catalysts with specific functionalities. In [3], Daniel Escorcia-Díaz et al. present an exhaustive review of the methods of deposition techniques to obtain functional composites. In [4], Nesterov et al. offer a rather interesting method for obtaining Co-Ni particles via precipitation in supercritical carbon dioxide. The use of engineering designs in the synthesis of nanomaterials is also capable of producing a synergistic effect, which is demonstrated in [5] using alloyed AuAg nanoparticles grafted on MoS_2_ nanoflowers or MoSe_2_@Graphene particles that were applied in hydrogen evolution reactions [6].

In addition to heterogeneous catalysis, nanocomposites also find applications in other catalytic processes, such as photocatalysis and electrocatalysis. Photocatalytic nanocomposites utilize the unique properties of nanoparticles, such as their ability to absorb light and generate charge carriers, to drive chemical reactions under light irradiation. For example, the visible-light active N-doped TiO_2_ photocatalyst composite was used in [7], and copper-modified titania-based photocatalysts were applied in [8] for efficient hydrogen production. Also, photocatalysts show promising results in the decomposition of organic dyes, such as methylene blue or rhodamine B, as shown in [9,10]. Electrocatalytic nanocomposites, on the other hand, facilitate electrochemical reactions by providing efficient charge transfer pathways, as shown in [11] for MnCo_2_O_4_/NiCo_2_O_4_/rGO and [12] for ZrO_2_/NiO/rGO systems.

Overall, the synthesis of nanocomposites and their applications in catalysis offer a wide range of possibilities for developing efficient and sustainable catalytic systems. The ability to tailor the composition, structure, and morphology of nanocomposites allows for the optimization of catalytic performance, opening up opportunities for advancements in areas such as energy generation, environmental remediation, and chemical synthesis.

In conclusion, I would like to thank all authors who contributed to this Special Issue, as well as the members of the *Nanomaterials* Editorial Board for their joint work and especially Greta Chang for her excellent help in preparing this Special Issue.
